# Supplementary material for: Patterns of Intron Gain and Loss in Fungi
Source: PLoS Biol. 2004 Nov 30;2(12):e422. doi: 10.1371/journal.pbio.0020422 (PMC532390; doi:10.1371/journal.pbio.0020422)
Supplement: Table S1 — Also available at http://genes.mit.edu/NielsenEtAl/. (4.3 MB ZIP). [file pbio.0020422.st001.zip › NielsenEtAl/html/818.html]

AN3684.1.NCU08652.1.MG10529.1.FG00573.1


```
 CLUSTAL W (1.82) Multiple Sequence Alignments - Introns Inserted


Sequence 1: MG10529.1	783 aa
Sequence 2: FG00573.1	665 aa
Sequence 3: NCU08652.1	715 aa
Sequence 4: AN3684.1	569 aa
Alignment Length: 835 aa
Number Identitical Residues: 71 aa
Alignment Score (without introns) 7083


MG10529.1 	-------------------MMPRSSFSDNPLLRVSRPVSACSRC~QSESLT~YR------
NCU08652.1	MSTLSFNWDDAHADLPSDSEATEEDLLDNPVLKVSRPVTACTRC1RSAKIK~CDGKLPAC
FG00573.1 	--------------------MPRPSFSHNPLLRVSRPVSACSRC1RAAKVK0CDGKLPAC
AN3684.1  	--------------------MSNSSFLDRPLLKVSRPVAACSRC~RTAKIR~CDGKLPAC
          	                     .. .: ..*:*:*****:**:** :: .:    .. .:.

MG10529.1 	-RRKRPAR2SNSQSQA1NDQFARGKERS2YVAALELRVEKLERRLKFAKSRKASMALHDV
NCU08652.1	TSCVKFGR~ESECMPI~SERFIRGREPN~YVRALEARVEKLEKQLHYARSHMSSQTQPGP
FG00573.1 	TACEKAGR~TNECSAA~NDQFARGKERS2YVAALELRIEKLEKRLQFAKSRKASVALHDT
AN3684.1  	SACERAGK~ADTCS-S~SDEFPRGKERS~YVGSLEAYCERLEKRAAELRERKRLLTGGEG
          	:   : .:  .      .:.* **:* . ** :**   *:**::    :.:    :    

MG10529.1 	DAPLAHSD-------------DRKGSMANIHAAIYRKAARQRENSDVNALVSDFGFL2SV
NCU08652.1	GPDPPTAPGPLDHGNDDEWGPDRRDSLDNIRVNVARRAARLHGDQEIEQVIASLSSV2TI
FG00573.1 	DASAFNVQQ-----------MDRRDSLALIRAAIHRKAAHKRETLDVNSLVSDFGFL2SV
AN3684.1  	G--------------------VVHENSITSASSVPATHAHSQEVSNIDDLVGEFGYL2SV
          	.                      : .       :    *: :   ::: ::..:. : ::

MG10529.1 	NATAQDFEPSVSNMTFARLLLAASTADPLPEPQTDANPPRDVARGLVKYYLDNIYALHPF
NCU08652.1	NPVSQEDHEKTR-LSLATIILASSTNKFVPQSRSVGLPPYDKARKIVDFYMSSVHQLYPA
FG00573.1 	NATTRDFEPISTNMTFARLVLAATINDALPEAPEACLPPRPTAHALVQHYMDNIYSLFPC
AN3684.1  	SATSRDFQGITSNTSFANLILAVSSGEQIPRSSPRPLPSRSEITPLIQHYFETFYVQLPF
          	...::: .    . ::* ::** :  . :*..     *.      ::..*:...:   * 

MG10529.1 	FPDVQLHSAVENLYHDDR-RHLKHHDCWLFWMVLAIASTAQSHSCNDEYYRNGVEYVTRA
NCU08652.1	FSRRIFNGLLEDIYRTSQ-RQFTAPEYWLFWMVLAIGSTAQSRQKDDSHYLDGLDYLACA
FG00573.1 	ISETALLTALDDMYDKNNSRHIQHSEYWMVYMVLAIGSISQSRRSNDTHYQDGLKYAARA
AN3684.1  	FLETSFWASVDSVYQNGA-HFAKPFDNWAVRMVLAMAYGSLSNSQLDVNHRNALSLVQEA
          	:    :   ::.:*  .  :     : * . ****:.  : *.   *  : :.:.    *

MG10529.1 	MTFADM~ALMPGKETQIRSLLLLTQYSLFDPAHFDSWHLIGFTCRAVIDLGFHQDPSSAE
NCU08652.1	LPFAQE~VLSPGNVKQIQSLLLLTLYSTYDPDHFDTWQILGFTCRAILDQGPFADPPEHH
FG00573.1 	VNHADE~ALAPGYVTQIQSLLLLTQYAMLDPAHFDSWHSIGFTARAIVDLGFHQDPPLSS
AN3684.1  	LQYTED0VWRP--------------------EFLLIWDCTRIIMR---------------
          	: .::  .  *                     .:  *.   :  *               

MG10529.1 	RIDKFKLNLRRKIFYCAYALDR2CIGMMSARAFSFTDDAVNVALPSMTGVGRIPSISGTI
NCU08652.1	GMSITDVKLRRRIFRSAYSLDR2AISLAHARAFSFADDAIPAEVRDALAD----------
FG00573.1 	VSDKSALDMRRKIFYCVYALDR2TISMVYARTFSFTDDTVNVAFPQASGNAGRGSASGAL
AN3684.1  	--------------------NM2ATSTALDRTLSFSDDSVNVAFPSSKLEK-------TY
          	                    :    .    *::**:**:: . . .            : 

MG10529.1 	KGPESSDPAILLFQLRQTQSHWYQTLVQSDPSVPLENAASYVWQMCHEMREWFESLPDGN
NCU08652.1	-GSSTAETSTYLYRLRRLQSTWYQSLFQGSPSHPLPDSTAFIWRMCHDMHAWFSTLPVS-
FG00573.1 	SGPQSADPALLLFQLRRAQSHWYQDLYQSG-SVPLQDPISYVWRMCLDMREWQDTLPNN-
AN3684.1  	IFSHSSEPAWNMVKIRRILSAAYQQKYFTTTDPSFQSPTP-TWVLYSQATEWFYNTPKN-
          	  . :::.:  : ::*:  *  **      .. .: .. .  * :  :   *  . * . 

MG10529.1 	EGLPTAIRQLFELELKYSYVYCIAPSSRAPTITSHARLLIFEYAVAYMNDVYDMVHPPDE
NCU08652.1	--HNPSIRQMLELEMYYSHVFCISPGIRSPHLSTYGELLIFEYAISYISGLCGLAD----
FG00573.1 	--LAPEIRQLFDQELRYSYVYCIAPSTRAPNITDYNRILIFEHSMAYLDTAYDIAQ----
AN3684.1  	--ISQVLAIRYHLEFLYTITVILAPSTRHLPPCDYTKLLLFNRCIDYVHQLHQILES---
          	      :    . *: *: .  ::*. *      : .:*:*: .: *:     : ..   

MG10529.1 	KPQIVLGEVKSSPSVSAWALLTYDQALRVYFMGSQFLAVMD--EMGETLLSEAPVAIPLS
NCU08652.1	-------------ASMNTALYTFHDVLRLFFVGTQFAAILRSDAADILLFSSHTISQSAS
FG00573.1 	-------------SGQNWGFFTYHDVMRIFFMASQFLAVLR--DAEDMLLSGMPVPIPIA
AN3684.1  	--------------QIRLHVMDSIEIQRVYQTIRRLFNIVN----QSFDVLMSPVPAAPQ
          	                   .    :  *::    ::  ::         .   .:. .  

MG10529.1 	LPGTAPPPPMPERR------GGDNLDRSLACVEKTIATLKKFGDRWNIANMLAQEFDRKS
NCU08652.1	QQGGVAPPPLPRKRMSDTTTTLPNIDRAITALGQIGKLLEWYGARWEDALSLGETFQMYS
FG00573.1 	RPGTAPAPPLPRRLQPQGMNGEDNLDRSLRCLDKVSQTLNIFGERWVDASHLRENFGLIS
AN3684.1  	VPEDCPKPPSLELED-----CLHCHERALECLNQAGNLLQYGARRWN-HHALSQEFQKLS
          	     . **  .             :*:: .: :    *:  . **     * : *   *

MG10529.1 	KDLCQRIEELKVLRSEQQEQQRQQLQQQQDQKPHHHQHQPHHQHNRSQTHVSTPHLQHQV
NCU08652.1	QDLMETLRGKKQQMGQRQVHQGHVQGQGHLQGQGQVQTQ---------------------
FG00573.1 	QEVVERLSERRQRRDTAREQYQSQYQ----------------------------------
AN3684.1  	APVRSILLPPAVTYAPTLG-----------------------------------------
          	  : . :                                                     

MG10529.1 	PPPPQYAMPMGQHSIPPMSPPLQQHMSPSAGHISPPARGGGIQMMGAPPPHANMMPGGYS
NCU08652.1	------------------------------GPGRAHTQGPG-------------------
FG00573.1 	------------------------------------SRG---------------------
AN3684.1  	------------------------------------------------------------
          	                                                            

MG10529.1 	AQPHMQQAHYHHQQPFPGQLSPLPPQVQQHPPVQHHTQPAEVNWAPVDINEIMRQGGGI-
NCU08652.1	-----------------GWENQQQQQQQQQHHHRQQSSGPQHRPPPMQQQTWPPGGYSLN
FG00573.1 	----------------------MPPQSQGGYQVQNSHQQEEMQWAGVDVAHMMRGGYPPQ
AN3684.1  	---------------------------SYMPEEPAILPPADFLYGGLNLQHSSPENHNYE
          	                           .            :     ::       .   .

MG10529.1 	---
NCU08652.1	NGQ
FG00573.1 	---
AN3684.1  	---
          	
```
